# Incidence of oral health in paediatric patients with disabilities: Sensory disorders and autism spectrum disorder. Systematic review II

**DOI:** 10.4317/jced.52923

**Published:** 2016-07-01

**Authors:** Begona Bartolomé-Villar, Mª Rosa Mourelle-Martínez, Montserrat Diéguez-Pérez, Manuel-Joaquín de Nova-García

**Affiliations:** 1 Stomatologist. Associate Professor of the Department of Dentistry, School of Biomedical Sciences, European University of Madrid; 2 Stomatologist. Contract Professor, PhD. School of Dentistry. Universidad Complutense de Madrid; 3 Dentist. Associate Professor. Department of Stomatology IV. School of Dentistry. Universidad Complutense de Madrid. Assistant Professor in the Dentistry Department. School of Biomedical Science. European University of Madrid; 4 Stomatologist. Tenured Professor of Paediatric Dentistry. School of Dentistry. Universidad Complutense de Madrid

## Abstract

**Introduction:**

We are currently witnessing an increase in the number of disabled patients, creating the need for knowledge of each of the pathologies and of the different oral and dental conditions they present, in order to achieve efficient management and treatment.

**Objectives:**

To analyse the existing scientific literature on the oral conditions of children with autism spectrum disorder (ASD) and children with sensory deficits (SD), in comparison with the healthy child population.

**Material and Methods:**

The bibliographic search was carried out in Pubmed/Medline, Scopus and Cochrane Library and included articles taking a sample of children between 0 and 18 years of age diagnosed with the abovementioned disorders and including at least one of the following oral hygiene conditions - oral hygiene, dental caries, malocclusion, oral habits, dental trauma, and gingival-periodontal status - comparing them with a healthy population.

**Results:**

A total of 10 articles were obtained for autism spectrum disorder and six for sensory deficits.

**Conclusions:**

Of all the variables studied, only the state of oral, gingival and/or periodontal hygiene can be considered worse in patients with ASD and SD, although we believe a larger number of research studies is needed to corroborate these results.

** Key words:**Oral health, dental caries, malocclusion, oral habits, dental trauma, oral hygiene, disabled child, autism, autism spectrum disorder, deaf, blind.

## Introduction

Children with sensory deficits and autism spectrum disorder pose a challenge to professionals in their dental treatment, fundamentally due to communication problems.

Patients with sensory deficits (SD) tend to present difficulties in socialisation, great dependence on their parents and/or carers, and increased fear and anxiety, which give rise to behaviour which dentists need to manage by following an appropriate protocol.

Autism spectrum disorder (ASD) encompasses a series of processes, all of which have a common denominator: a lack of affectivity and social relation, monotonous and stereotyped activity, which entail limited and repetitive behaviours. Many of these children also present language disorders, mental retardation and sensory-perceptive problems (1).

The dentist should know what conditions to expect in each patient and which techniques are the most appropriate for managing them: basic techniques (communication, distraction, imitation, desensitisation), physical techniques (restraint by the professional/assistant/parents or using specialised devices) and advanced techniques (nitrous oxide, sedation or even general anaesthesia). These techniques should be individualised, keeping in mind not only the patient’s disorder, but also its seriousness, to gain an idea of the degree of cooperation that can be obtained. In addition, we should analyse the patient’s oral pathology, since the possibility of long or complex treatments may help us select the most advisable technique (2).

Certain disorders have been described as being more prevalent in association with these disorders - malocclusions, enamel hypoplasia, gingivitis and/or periodontal disease, parafunctional habits (bruxism) and deleterious habits (mouth breathing, tongue thrusting, rumination) - as well as a higher incidence of dental trauma, owing either to accidents or to self-imposed injuries. However, not all the studies obtain similar results and some are even contradictory when certain pathologies are analysed. Hence the objective of this systematic review is to find whether there is scientific evidence that certain oral and dental disorders occur with greater frequency in the group selected.

## Objectives

To analyse the existing scientific literature on the oral conditions of children with autism spectrum disorder (ASD) and children with sensory impairments, in comparison with healthy children with no medical, psychological or motor system pathology.

## V

The P.I.C.O (Patient, Intervention, Comparison and Outcome) question asked prior to the search was: do children with sensory deficits and autism spectrum disorder present a greater oral and dental pathology than healthy children?

The selection of articles was made based on the following criteria:

- Study population: Clinical studies on humans which include and specifically analyse the disabled population groups selected: those with sensory deficits and autistic spectrum disorder.

- Intervention: The oral and dental examination was considered the intervention that should be present in all the studies. At least one quantitative measure of the state of oral health.

- Comparison: The study should include a control population without pathology.

-Results: The primary result was to observe whether the disabled population presented conditions of oral health which were better or worse compared to the control population, analysing at least one of the following variables: dental caries, malocclusion, oral habits, dental trauma and oral hygiene.

The process was based on certain items proposed by the guidelines of the PRISMA statement:

• Search strategy:

A search was carried out for scientific articles published in indexed journals using the following databases and information sources: PubMed/ Medline, Scopus and Cochrane Library. The keywords used were: “oral health”, “dental caries”, “malocclusion, “oral habits”, “dental trauma”, “oral hygiene” and “disabled child*,” to which a specific keyword was added for each disorder selected: “autism”, or “autism spectrum disorder,” “deaf,” or “blind.” Each search was complemented by combining the different keywords referring to oral conditions with the terms “disabled child*,” and afterwards with the keyword of the particular disorder being analysed.

• Criteria for the selection of articles:

Studies which met the following criteria were included:

1. Type of publication: original studies in indexed journals.

2. Year of publication: from 2000 to 2015.

3. Language: no limitations.

4. Design of studies: randomised clinical studies, prospective and retrospective clinical studies.

5. Study population: sensory deficits and autism spectrum disorders with an age range between 0 and 18 years.

6. Comparison: Presence of a control group in the study.

7. Study variables: A quantitative measure of the state of oral health.

Studies that did not meet the following criteria were excluded:

1. Studies whose sample size was smaller than 10 patients.

2. Bibliographic reviews, meta-analyses, presentations to congresses, editorials, opinion articles or reports of isolated cases.

• Extraction of data, variables and presentation of findings

The articles were selected independently by two reviewers, each covering one of the disabilities (autism spectrum disorder, sensory deficits). In a first selection, any articles that appeared in all three databases selected were excluded. Subsequently, potentially relevant articles were screened, eliminating.

- Those that, after reading the title and/or abstract, were not considered appropriate, according to the inclusion criteria.

- Articles which, upon reading of the complete text, did not meet the established norms:

- Articles that analysed the variables selected, but whose results were not obtained from clinical examination (data obtained through interviews, questionnaires given to parents or caregivers.)

- Articles referring to the disabled population in general without specifying in the results the actual disorder.

- Articles which were inappropriate because of the age group studied or because they lacked a healthy control group.

The results of the variables analysed were reported independently by each reviewer in a data collection table designed for that purpose. The data collected were: first author, year of publication and country where the study was carried out. Characteristics of the disabled group and of the control group. Study variables and methodology. Results obtained.

• Evaluation of the articles selected:

A scoring table was designed, based on the Newcastle-Ottawa Quality Assessment Scale. The information collected in the table referred to.

1. Selection. Specific diagnosis of the disability and the representativeness of the sample.

2. Comparability. Evaluation of whether the control group presents characteristics similar to the study group (sample size, age, sex, socio-economic characteristics…). To avoid bias, the comparison groups should be as similar as possible. A positive evaluation is given if they meet at least two of the characteristics.

3. Study variables. Information about the oral health of the sample and about:

a. Characteristics of the examiner or examiners: the presence of a consistency test in the case of more than one, or an intra-examiner reliability test in cases where there is only one.

b. Material and diagnostic method for the variables studied. A positive evaluation is given if it is common to both study groups.

c. Criteria for evaluation of the variables studied.

In order to be included, the articles had to have a minimum of three points (1: disorder selected in a population of 0-18 years of age, 2: comparison with a healthy control group, 3: inclusion in the study of at least one of the variables selected). The score increased as more criteria included in each section were met ( Table 1).

**Table 1:**
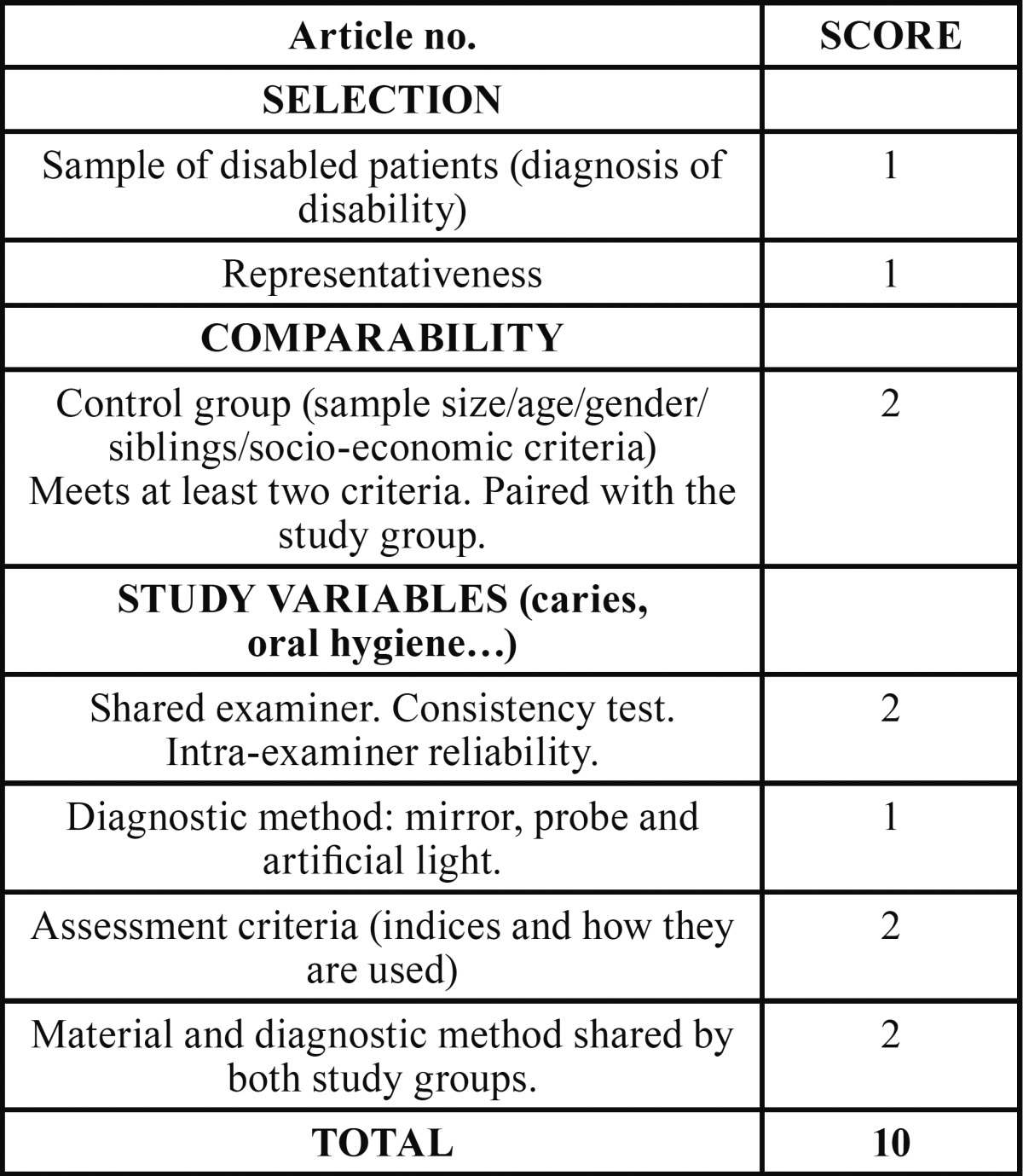
Table on assessment of selected articles.

Subsequently, at a joint meeting, the searches were presented and the articles that met the requirements were selected to form part of this review.

## Results

**Figure 1:**
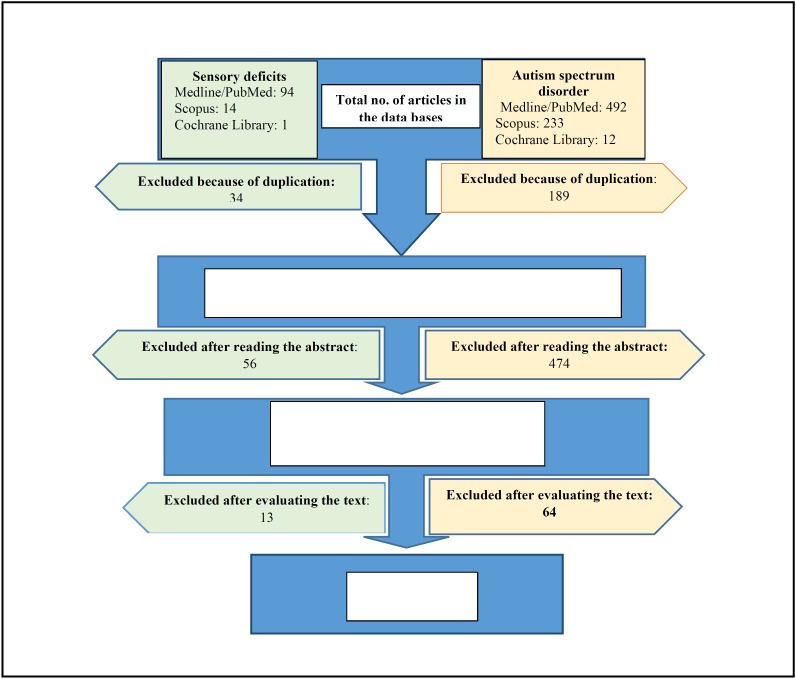
Flow diagram for the two reviews.

• Search results:

Figure 1 shows the flow chart. In the electronic search, a total of 846 articles were found: 586 in Medline/Pubmed, 247 in Scopus and 13 in Cochrane Library. After comparing the articles obtained, any articles duplicated were excluded, reducing the number to 530. Of the potentially relevant articles, a first reading was done of the title and/or abstract to see their suitability, and any articles not considered appropriate were excluded. This reduced the sample to 75 in Medline/Pubmed, 18 in Scopus and 0 articles in Cochrane.

After reading the complete texts of the 93 articles, the following were excluded:

- Articles in which the sample was insufficient or the age group higher than the one selected: 25 (21 on ADS, 4 on SD).

- Not specific to sensory/autistic disorder: 21 (19 on ASD, 2 on SD).

- No control population included: 20 (17 on ADS, 3 on SD)

- Inclusion of an unhealthy control population: 3 (1 on ADS, 2 on SD)

- Results obtained from questionnaires: 8 (6 o ADS, 2 on SD)

The total study sample that met our inclusion criteria consisted of 6 articles on sensory deficits and 10 on autism spectrum disorder.

• Study population:

In this review, a total of 3,739 patients were examined: 683 with visual impairment, 200 with hearing disorders, 628 with autism spectrum disorder and 2,228 controls. The study with the smallest sample was done by Fahlvik-Planefeldt and Herrström, with 20 autistic children and 20 controls (3), while the largest sample size corresponds to the study done by Ameer *et al.*, on 300 patients with visual and hearing impairment, comparing them with 150 healthy patients (4). Of the 6 studies reviewed on sensory deficits, in only two of them was gender specified (5,6), while in the ones referring to autism disorder, in all of them the number of children was specified according to gender (generally, the percentage of males is higher in the population under study, since autism disorder is more prevalent in this sex), except in two that do not mention gender (7,8).

• Intervention:

All the children in this review were given an intraoral examination. In the eight articles, this was performed at schools, centres or institutions (5,9-15); in two, the examination was done in dental chairs/clinics (3,16) and in the remaining six articles, the place where the patients were examined was not specified. In the majority of cases, one/two examiners intervened and only Ameer *et al.*, in their study of children with hearing and visual impairment, used three calibrated examiners, while Rai *et al.* (7) and Bassoukou *et al.* (8) did not specify in their material and methods how many examiners were involved. The study by Fahlvik-Planefeldt and Herrström carried out in 2001 is the only one that mentions the possibility of taking bitewing x-rays as a complement to clinical examination, if this were considered necessary for the diagnosis, or if the patient showed a high degree of cooperation (3).

The oral conditions analysed vary, although the majority of the studies examine dental caries and periodontal status. In 11 articles (nine of which refer to the autism spectrum and two to sensory impairments), the prevalence and/or incidence of dental caries is evaluated mainly using the CAOD/caod indices, following WHO criteria (3,7-14,16,17); in 10 articles (3,4,6,7,9,11,12,14-16), the state of oral hygiene is examined, either through the plaque index or the simplified oral hygiene index; two articles evaluate the dental restoration index (12,16); in nine, the gingival/periodontal condition (3,4,6,9,12,13,15,16,18), using the gingival index, bleeding and plaque on probing or CPITN; in two, the existence of bruxism as a parafunctional habit (3,9); in two, the need for orthodontic treatment (3,18) and in three, the prevalence of dental trauma (5,9,14). Additionally, other conditions were studied which are not analysed in this paper, such as the presence of lesions in soft tissue (9); the existence of self-injurious habits (11), the degree of cooperation of the patient, according to the Frankl scale (3,9), and the saliva pH (7,8). These last two articles also evaluated the concentration of salivary antioxidants (in the first article) and the saliva buffer capacity (in the second).

• Results of the intervention:

- Prevalence and incidence of dental caries:

There are discrepancies about the results found in the different studies regarding dental caries. Three studies show no significant differences between children with ASD and the control group (7-9), five studies show a greater incidence of dental caries among children with the three disabilities, three report statistically significant differences (11,16,17), and one finds none (12) or does not make specific reference to them (14). Fahlvik-Planefeldt and Herrström observed a lower number of caries in autistic children (50% with caries compared to 70% in the control group) but without specifying the significance (3), while Namal *et al.* (10) and Fakroom *et al.* (13) did observe significant differences, with the former author even affirming that not having autism supposed a 3.99 times greater predisposition to dental caries.

- Oral hygiene:

Of the ten studies that examined the oral hygiene index, all except one (3) found a worse state of oral hygiene in children with the three disabilities analysed, with the differences being statistically significant in six of them (4,7,9,11,12,15). Ameer *et al.* found the highest scores for plaque (96%) in the groups with visual impairment, followed by the patients with hearing impairment, with 9.7% (4). Only the study by Fahlvik-Planefeldt and Herrström showed no differences, with oral hygiene being acceptable in both groups (3). Jaber did not indicate any significance, observing that 59% of the control group presented good oral hygiene, compared to only 3.3% of the children with ASD (16); and Reddy *et al.* found poorer oral hygiene in the group of children with visual impairment, although without specifying the significance (14).

- Gingival index and periodontal status:

The study by Fahlvik-Planefeldt y Herrström was the only one that did not find significant differences in the evaluation of the gingival status of children with ASD and the control group (3). Al-Maweri *et al.* (12) and Fakroon *et al.* (13) observed a higher gingival index for children with ASD, although they do not point out the significance. The latter study found that more than half of these children need scaling and 38.2% presented gingival inflammation (more than double that of the control group). This worse periodonal status was seen to be statistically significant in two studies of the autism spectrum disorder (9,18) and in the three referring to sensory impairment, fundamentally in those with visual deficits (4,6,15) although Jain *et al.* observed differences only in the bleeding index (6).

- Dental traumas:

The study by Bhat *et al.* of a population with visual impairment shows statistically significant differences with respect to fractures in anterior teeth (*p*= 0.001), with the percentage affected being 32.5% in the children with visual disability and 9.6% in the healthy population. An overjet of more than 3.5 mm significantly increases the risk of dental trauma in the group with visual disability (70.4%) with respect to the control group (46.3%) (5). Reddy *et al.* found a greater frequency of trauma in the group with visual impairment, although they did not specify whether those differences were statistically significant (14).

- Habits:

A larger number of children with wear indicating bruxism was observed by El Khabit *et al.* (9). Richa *et al.* (11) and Al-Maweri *et al.* (12) observed a greater frequency of self-inflicted injury in soft tissues and self-injurious habits in autistic patients.

- Need for orthodontic treatment:

Only two studies referring to ASD evaluated the need for orthodontic treatment. Neither found differences between the two groups, although Fahlvik-Planefeldt and Herrström pointed out that a greater percentage of the children with autism (60%) needed treatment, compared to 40% in the control group (3). These data agree with those of Luppanapornlarp *et al.*, who found a larger number of malocclusion disorders (60%) in autistic children, fundamentally missing teeth, diastemata, inverted overjets, open bites and class II molars (18).

- Others:

Two studies show statistically significant differences in the degree of cooperation, with ASD patients presenting negative or definitely negative behaviour, according to the Frankl scale (3,9).

Two other articles included a study of saliva pH, which did not find statistically significant differences, although Rai *et al.* indicated that there was significance in the concentration of salivary antioxidants, which was lower in children with ASD (7). Bassoukou *et al.* found that the children with autism between 9 and 13 years of age presented a lower saliva buffer capacity with respect to the control groups (8).

All the results obtained are shown schematically in tables  Table 2 and  Table 3.

**Table 2:**
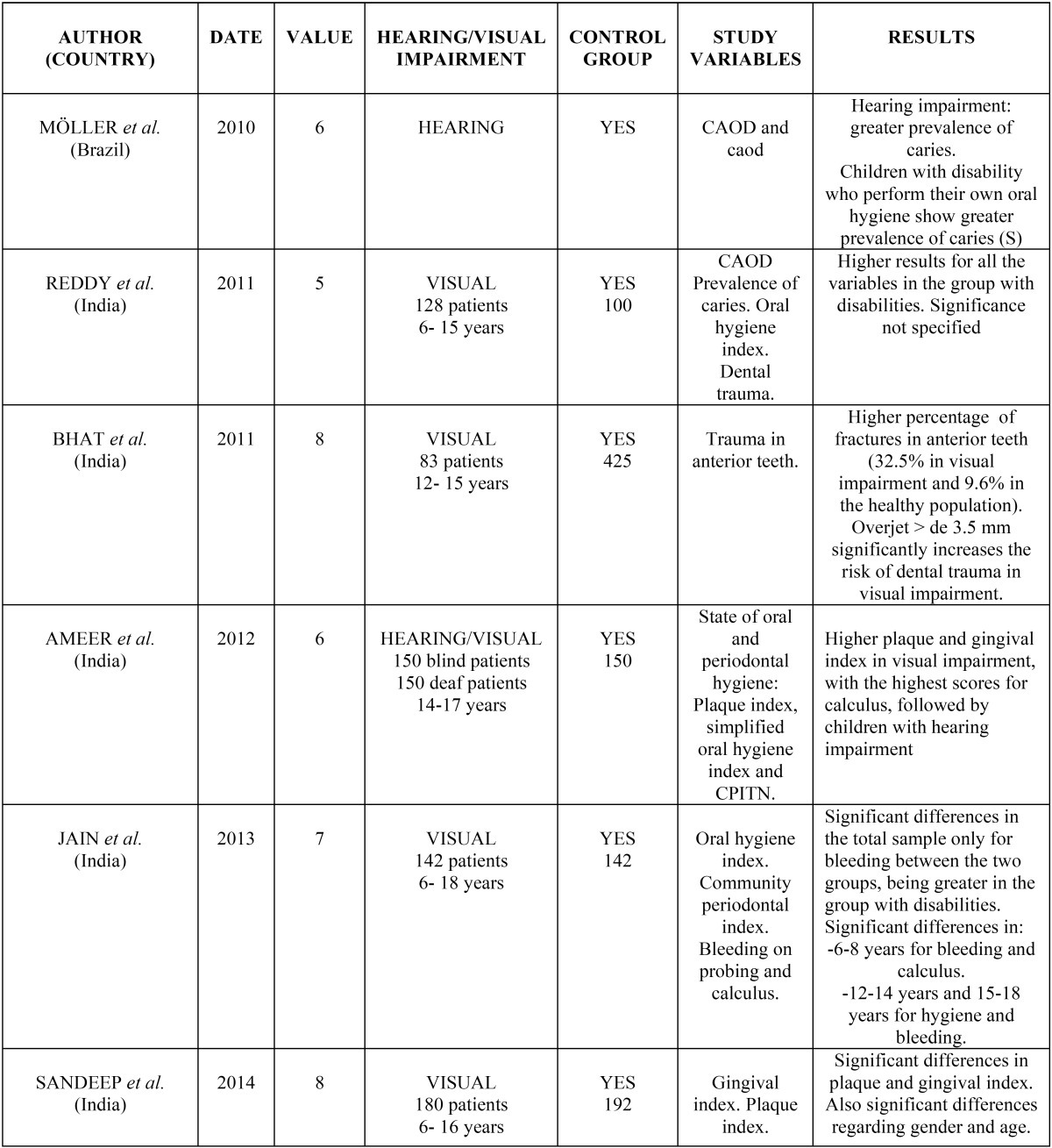
Results of the analysis for sensory déficits.

**Table 3:**
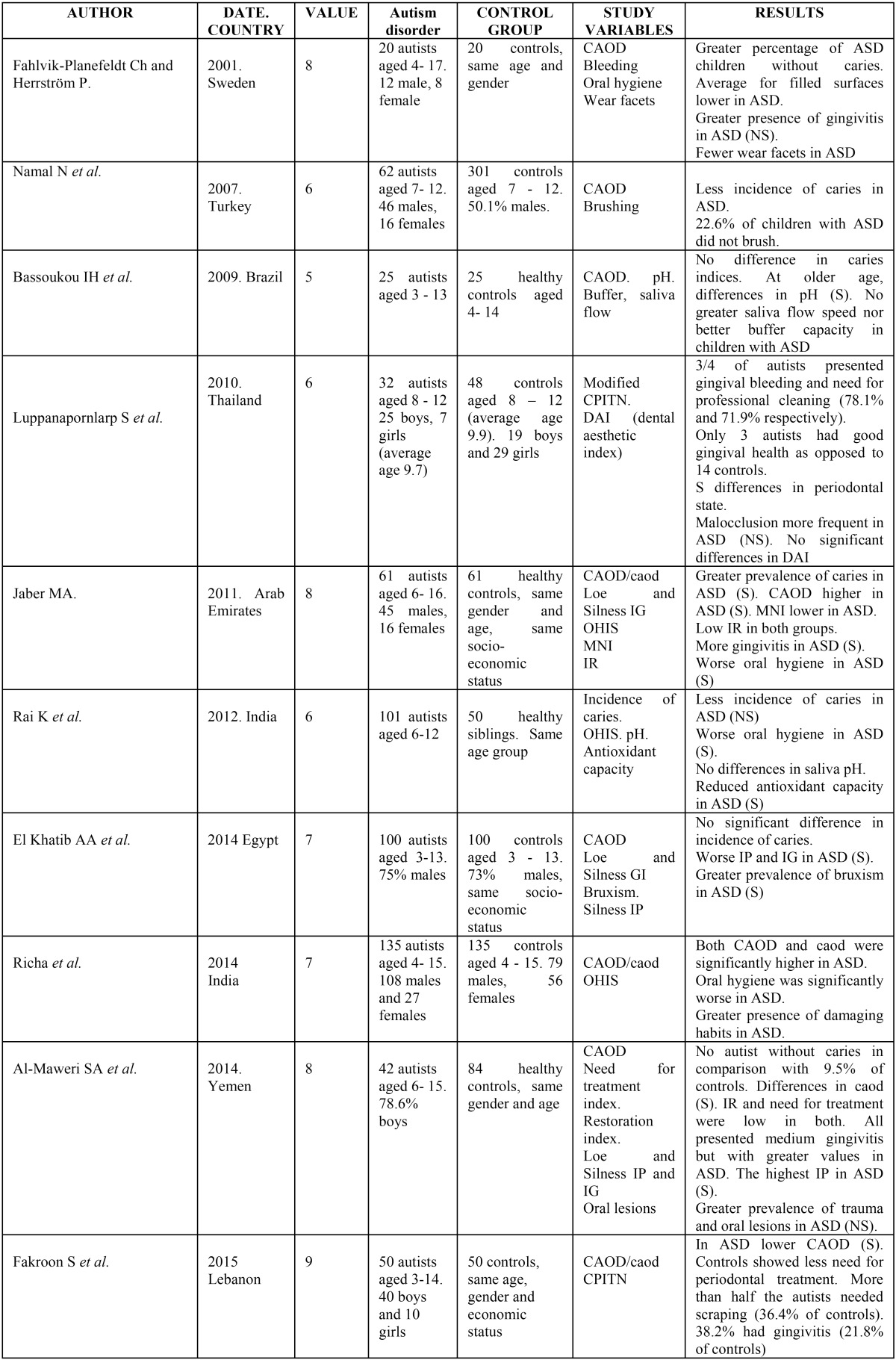
Results of analysis of articles for Autism spectrum disorder.

## Discussion

In spite of the increased number of children with special needs, especially those with neurosensory disorders, few research studies have been done on the state of their oral health, comparing the paediatric population with these disabilities to healthy control groups. In our review of the literature on children with sensory disorders and autistic spectrum disorder, we found only 10 papers on ASD and six on paediatric patients with visual and/or hearing impairments. Some papers included in this review do not specify the instruments used, the characteristics of the intraoral examination and where it was performed, or the characteristics of the control population. We established a scale to evaluate the characteristics of samples, methodologies and clinical interventions, with the result that the majority of the articles were deficient in at least one of the areas. The median score was 6.6 for the articles referring to sensory disorders and 7 points out of 10 for the ones analysing oral and dental parameters in autistic patients.

There is no unanimity regarding the incidence of dental caries in children with ASD, compared to the general population. Two of the studies showed a similar prevalence (8,9), four a lower incidence (3,7,10,13) and three a higher one (11,12,16). The study by Al-Maweri *et al.* did not find any child without caries among the 42 autistic children examined (12). The opposite was found in children with sensory deficits, since the only two papers included (14,17) found an increase in CAOD and coad, especially in patients who perform their own oral and dental hygiene (17).

All the studies analysed point to a worse state of oral and gingival hygiene reflected in the plaque index, bleeding or the community periodontal index (CPITN); only Fahlvik-Planefeldt and Herrström did not find differences in the gingival index between the control group and the children with ASD (3). This result could be due to the difficulty that these patients have in being autonomous, as they generally depend on parents, teachers or carers, who must be motivated and trained in order to suitably carry out oral health programmes.

An increase among children diagnosed with ASD of other disorders, such as malocclusion (3,18) or para-functional (9) or self-injurious (11,12) habits were found, although without statistically significant differences with respect to the control population. Other manifestations referring to the amount of saliva or its buffer capacity were not found to be affected in autistic children (7,8) although a lower amount of antioxidant elements were found in their saliva (7).

Reddy *et al.* (14) and Bhat *et al.* (5) observed a higher prevalence of trauma in patients with sensory impairment (mainly in patients with visual impairment), with the differences being significant in the latter study.

## Conclusions

In this review, we did not find differences in the incidence of dental caries or the presence of malocclusions and oral habits in children with sensory impairment and/or diagnosed with autism spectrum disorder. Worse oral hygiene and a more deficient gingival/periodontal state are confirmed in both disabilities, compared to the healthy paediatric population, as well as an increase in the frequency of traumas in patients with sensory impairment. However, we believe that in order to corroborate these results, it would be advisable to carry out a greater number of studies with larger sample sizes to analyse the differences in paediatric oral and dental health between the selected group and the control population.
